# Editorial: *NAR Genomics and Bioinformatics*: a new journal for reproducible genomics in the Big Data era

**DOI:** 10.1093/nargab/lqz001

**Published:** 2019-04-17

**Authors:** Cedric Notredame

**Affiliations:** 1 Centre for Genomic Regulation (CRG), The Barcelona Institute of Science and Technology, Dr. Aiguader 88, Barcelona 08003, Spain; 2 Universitat Pompeu Fabra (UPF), Barcelona, Spain

It is my pleasure and privilege to introduce to you our new scientific journal, *NAR Genomics and Bioinformatics*. This new title is meant to respond to the growing need for methods combining sequencing and computerized analysis. With high-throughput procedures delivering more and more data, our community is now under pressure to keep up by developing more efficient and more complex *in-silico* methods while making sure the associated analyses remain reproducible. For many years, NAR has been the right place to publish such contributions. But the times are changing and the reliance of the newest *in-silico* methods on increasingly diverse theoretical foundations is driving them away from NAR’s original audience. Genomics, and -omics analysis as a whole, are now highly dependent on IT technologies, machine learning and statistics. Many of these needs connect biology to the much broader picture of *Big Data* analysis. For instance, it is a striking observation that some of our most widely used representation techniques such as t-SNE or PCA are not particularly specific to biology but shared across all disciplines that make use of *Data Science* (e.g. economics, sociology or resource management). These increasingly complex computational procedures have also contributed towards identifying reproducibility as a major issue faced by modern science. There exists a growing sense that some of the basic principles underlying the publishing and dissemination of scientific results will need to be re-thought and somehow adapted. For all these reasons, we feel that the time has come to provide our community with a new scientific venue. *NAR Genomics and Bioinformatics* has been designed to accommodate the novel combination of skills required by genomics and to help it face the forthcoming challenges of reproducibility. We will do so by remaining true to the original values upheld by NAR over the last five decades. Our focus will be on high quality science, unassuming albeit carefully selected for long-term impact. To that effect, we welcome contributions from all fields of research connected to genomics. We hope the community will share our excitement and join us in this adventure at a time when biology is undergoing a dramatic transition towards data science.

Such evolutions are hardly unusual in science and, to be fair, when it comes to hiring other's brains, biology is possibly one of the most opportunistic scientific disciplines ever explored by mankind. Over the last two centuries the basic skills required for a biologist to collect data—wherever and however—have kept shifting. Darwin's generation required fearlessness, marksmanship and drawing skills. In the early 20th century, Fisher cranked modern statistics up one notch and forced wide open the doors of population genetics and evolutionary biology. Mathematics had become an essential skill for biology. In the ’30s electron-microscopy and X-ray crystallography opened another dimension and made us dependent on the skills of physicists—with cryo-EM being the latest incarnation of their talents (1). Count a couple more decades and here come cloning and sequencing—two tasks unthinkable without the contribution of chemists such as Fred Sanger or Werner Arber. I guess this makes the point that modern biology, born out of the concept of evolution, does just like the object of its studies: it adapts out of chance and necessity. One of the latest of these adaptations has been high throughput sequencing and its long pedigree of next next-generations. The ubiquitous log-scale graph showing the drop of sequencing costs screams a simple message: the time has come, once again, for biology to adapt and transition across disciplines. The new kids on the block are machine learning scientists and IT technologists, coming to the rescue of the first migrant wave of computer scientists, the ones who came to visit biology in the ’80s and ’90s. These newcomers will add their skills to the already formidable arsenal of interdisciplinary biology. Such expansions are never neutral. They generate new common vocabularies, reveal new needs, unlock new directions and allow new encounters, as dramatically illustrated by the recent excursion of Google into structural biology (2).

While the crossover between deep learning and structural biology is rather new, the idea of interdisciplinary study pushing fields of research into unchartered territories has been here for a while. In fact, it is exactly the point made on the first page of NAR on the day of its launch (Figure 1) in January 1974. From Day One, NAR branded itself as an interdisciplinary journal for anyone interested in the ‘physical, chemical, biochemical, biological, or medical properties of nucleic acids’. Forty-five years later, NAR has become a fixture of scientific life, a place where good, honest and dependable science gets published and relied upon. These carefully crafted first few words define an important legacy, a set of values proudly embraced by *NAR Genomics and Bioinformatics*. Our goals are the same but come directed at different crowds. We want to offer a space of exchange and mutual nurture for biologists, computer scientists, geneticists, mathematicians and, in general, anyone willing to contribute to the formidable new adventure of genomics and bioinformatics whose new take on biology is slowly shaping on our screens.

**Figure 1.**
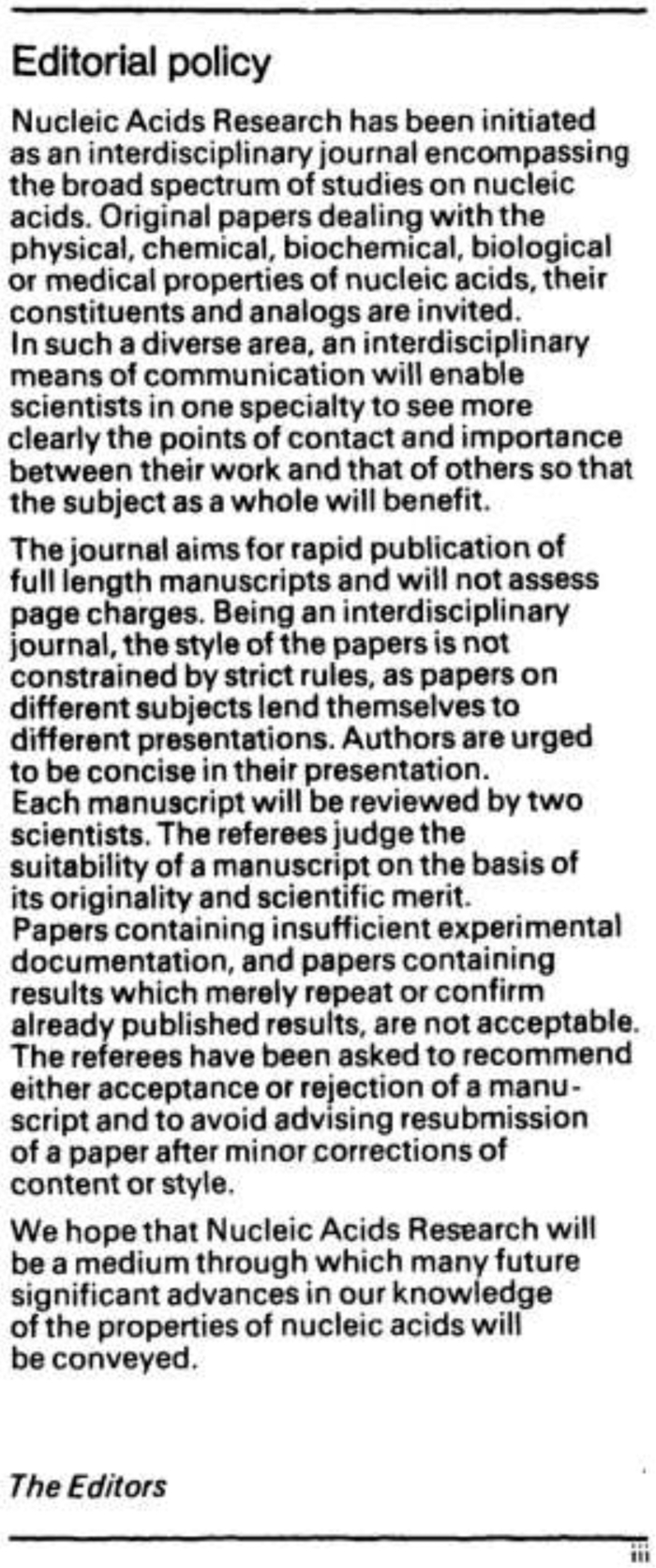
Front matter of the first issue of *Nucleic Acids Research* as it appeared in January 1974.

And the challenges are many! I don’t think I am being careless by predicting that the current data deluge will change most things we think we know in biology. Imagine what will come out of the 66.000 genomes project announced just a few months ago (3). Looking back in history, there never was a single instance when new ways of acquiring data did not lead to the re-foundation of theoretical principles. Just see how Newtonian mechanics was blown away by astrophysics and atomic measurements. Data has this effect on theories, and biology is no exception. Biology had its own share of data-driven revolutions, and every time we take our kids to one of these beautiful art deco Natural History museums scattered around the world, we pay tribute to the first data expansion era of the field. It incidentally peaked the year Darwin's second *HMS Beagle* expedition sailed away (Figure 2). Given how much was achieved with so little data, it is hard not to be optimistic about what is to come next. It took 20 years to digest the *HMS Beagle* data into *On the Origin of Species*, and we certainly will not have it easier. With so many novel repurposing possibilities, from natural conservation to cancer treatment, the theory of evolution has never been so data-hungry. One of our tasks, as a community, is to make sure that Darwin's rocket never runs out of fuel. We will achieve this by feeding the virtuous cycle linking data production and novel hypothesis.

**Figure 2.**
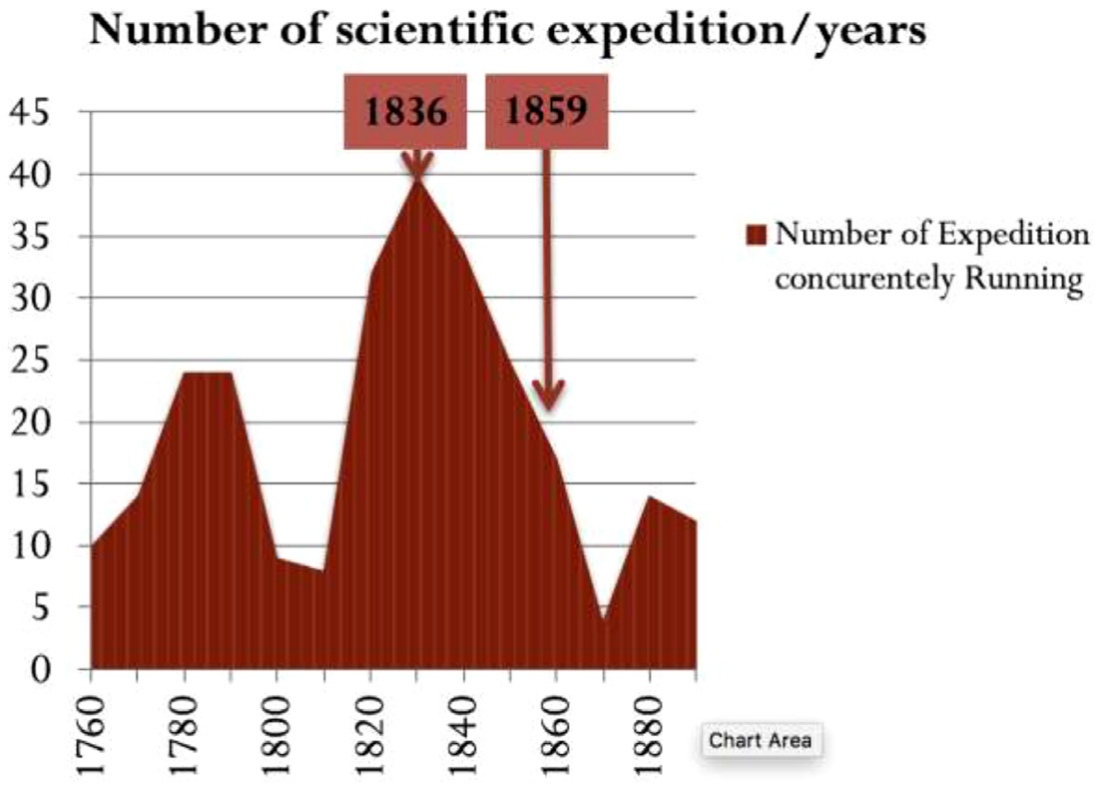
Number of scientific expeditions concurrently running (Source Wikipedia: https://en.wikipedia.org/wiki/European_and_American_voyages_ of_scientific_exploration). The total duration was obtained by subtracting the start date from the return after excluding expeditions with no return date. The date 1836 corresponds to the second voyage of *HMS Beagle* and 1859 is the date of publication of *On the Origin of Species* by Charles Darwin.

Keeping this flow steady is not a simple task as it requires a perfect balance between data and analysis—a real challenge at a time when data doubles roughly twice faster than computational power (4). This differential is the 800 petabytes gorilla in the room. If we do not want King Kong to start hopping onto the NCBI or the EBI, then something has to give. Until quantum computers deliver—a lot of uncertainty there—we will have to run faster than data with our smarter algorithms or turn off the sequencers. I would argue here that bioinformatics is anything that will allow us *not to turn off the sequencers* while genomics is anything that makes the sequencing machines run faster. If you think there is some kind of tension here, well … you are not alone ….

The question of how to define bioinformatics will sound familiar to many scientists of my generation. As a biologist I grew up with the notion that experimental data was scarce. There were few sequences, few structures and, in general, little of anything you could measure. All databases were sent to you on a CD every couple of months. With data out of reach, predictions were the next best alternative and it is no coincidence that the first generation of bioinformaticians embarked on the ambitious journey of predicting *everything*. And I really mean *everything*, the genes, their regulation, the structure of the proteins, their interaction with proteins, and with DNA. All this from first principles of course. There were a few successes here and there, like protein and RNA secondary structures for instance. But that's about it. While bioinformaticians were busy designing and benchmarking their algorithms, biologists became restless and came up with a new way of sequencing. The human genome project had just started, and it was becoming clear that speeding things up was not merely optional …. This was a big transition, and I would argue that this is when molecular biology and computer science became irrevocably entangled. It is probably no coincidence that this corresponds to the time when the computational feasibility of gunshot sequencing was demonstrated and hidden Markov modelling repurposed from speech recognition to biological sequence analysis. This combination of computational and technological breakthrough made the genomics we know today possible. A simple glance at the latest developments, from single cell transcriptomics all the way to Hi-Seq analysis confirms that the future of our discipline will be split between CPUs and Eppendorf tubes.

This ocean of possibilities brings just as many issues. Reproducibility has been under scrutiny for quite some time and whatever shift is to take place over these next years, business as usual is not on the menu. The community awareness of these issues has materialized in massive collective efforts such as ELIXIR or the Global Alliance for Genomics and Health. This spells good news and the time is getting closer when full computational reproducibility based on seamless interoperability is to be taken for granted. The instruments of this long needed revolution will be widely accepted standards allowing each letter of the FAIR principle (Findable, Accessible, Interoperable, Re-usable) to guide our work. This aspect is certainly one of the points in which we will make sure that *NAR Genomics and Bioinformatics* acts as a worthy contributor. We will have to be both stubborn and pragmatic, keeping in mind that the road leading to most established standards is littered with unexpected failures—think Betamax if you are over 50 …. As a scientist my take on this is very simple. Standards only make sense when they unlock new possibilities. It has to be a bottom up process, driven by a simple and effective Darwinian principle in which communities of scientists and their needs are the selective pressure. Such processes can crystalize very fast and our goal as a journal will be to help this happen. With strict and realistic constraints on how tools and data should be deposited in public repositories, we will insure that your work becomes as widely available, usable and—most importantly—re-usable as possible. This will be our commitment and our measure of success. It will be good for the community to have better tools and more trustworthy results and it will be good for the authors whose work will have increased visibility.

This adventure is just starting and it is already one of the most exciting endeavours I have encountered in my scientific career. Fortunately, I am not alone in this and I want to thank the many outstanding scientists who have agreed to join the board. I can easily measure how privileged I am to be in such company. Thanks to their expertise our journal will cover genomics and bioinformatics in the broadest current and future acceptance of these terms. Their help and guidance will be instrumental to insuring your research reaches its widest intended audience.

## References

[1] Baker M. Cryo-electron microscopy shapes up. Nature. 2018; 561:565–567. 30254359 10.1038/d41586-018-06791-6

[2] Service R.F. Google's DeepMind aces protein folding. Science | AAAS. 2018; doi:10.1126/science.aaw2747

[3] Stokstad E. Researchers launch plan to sequence 66,000 species in the United Kingdom. But that's just a start. Science | AAAS. 2018; doi:10.1126/science.aav9295

[4] Langmead B., Nellore A. Cloud computing for genomic data analysis and collaboration. Nat. Rev. Genet. 2018; 19:325 10.1038/nrg.2018.8 29430012

